# Crosstalk between Oxidative Stress and SIRT1: Impact on the Aging Process

**DOI:** 10.3390/ijms14023834

**Published:** 2013-02-11

**Authors:** Antero Salminen, Kai Kaarniranta, Anu Kauppinen

**Affiliations:** 1 Department of Neurology, Institute of Clinical Medicine, University of Eastern Finland, P.O. Box 1627, FIN-70211 Kuopio, Finland; 2 Department of Neurology, Kuopio University Hospital, P.O. Box 1777, FIN-70211 Kuopio, Finland; 3 Department of Ophthalmology, Institute of Clinical Medicine, University of Eastern Finland, P.O. Box 1627, FIN-70211 Kuopio, Finland; E-Mails: kai.kaarniranta@uef.fi (K.K.); anu.kauppinen@uef.fi (A.K.); 4 Department of Ophthalmology, Kuopio University Hospital, P.O. Box 1777, FIN-70211 Kuopio, Finland

**Keywords:** ageing, autophagy, oxidative stress, inflammasome, NF-κB, ROS, SIRT1

## Abstract

Increased oxidative stress has been associated with the aging process. However, recent studies have revealed that a low-level oxidative stress can even extend the lifespan of organisms. Reactive oxygen species (ROS) are important signaling molecules, e.g., being required for autophagic degradation. SIRT1, a class III protein deacetylase, is a crucial cellular survival protein, which is also involved in combatting oxidative stress. For instance, SIRT1 can stimulate the expression of antioxidants via the FoxO pathways. Moreover, in contrast to ROS, SIRT1 inhibits NF-κB signaling which is a major inducer of inflammatory responses, e.g., with inflammasome pathway. Recent studies have demonstrated that an increased level of ROS can both directly and indirectly control the activity of SIRT1 enzyme. For instance, ROS can inhibit SIRT1 activity by evoking oxidative modifications on its cysteine residues. Decreased activity of SIRT1 enhances the NF-κB signaling, which supports inflammatory responses. This crosstalk between the SIRT1 and ROS signaling provokes in a context-dependent manner a decline in autophagy and a low-grade inflammatory phenotype, both being common hallmarks of ageing. We will review the major mechanisms controlling the signaling balance between the ROS production and SIRT1 activity emphasizing that this crosstalk has a crucial role in the regulation of the aging process.

## 1. Introduction

As an organism ages, more and more errors occur in the cellular homeostasis system, e.g., in energy metabolic regulation and protein quality control [1–4]. Mitochondrial defects are one crucial component of the aging process and several age-related diseases. Mitochondrial impairments increase the production of reactive oxygen species (ROS) and the appearance of oxidative stress, a condition that increases with aging. Mitochondrial disturbances lead to deteriorations in protein quality control, especially to the decline in autophagic degradation with aging [1,2,5]. The increased ROS production enhances the accumulation of aberrant protein aggregates and dysfunctional organelles, which activate inflammasomes, cellular receptor systems dealing with danger-associated molecular patterns (DAMPs) [6–9]. On the other hand, there are multiple survival mechanisms, which can combat stress-related dangers by improving the efficiency of housekeeping, e.g., autophagic cleansing potency. These actions support healthspan and thus can extend lifespan. One of these longevity factors is SIRT1, a class III protein deacetylase. This enzyme can regulate several survival functions by deacetylating not only histones but also many crucial transcription factors e.g., those controlling autophagy and ROS production [10–13]. Interestingly, it seems that oxidative stress and the increased levels of ROS, in turn, can control the activity of SIRT1. We will review the major mechanisms involved in the crosstalk between SIRT1 and ROS and examine how this context-dependent balance governs both autophagy and inflammatory responses, which are key factors in the regulation of the aging process.

## 2. Falling of the Aging Dogmas

### 2.1. Free Radical Theory of Aging

In 1956, Denham Harman [14] proposed his theory on the role of free radicals as the cause of the aging process. He refined his theory later, e.g., implicating mitochondrial function in the production of free radicals and the extension of life span [15]. Because of unpaired electrons, free radicals are very reactive in cellular milieu attacking cell constituents, thus causing damage. In cellular context, the most common reactive oxygen species (ROS) are superoxide anion, hydrogen peroxide and hydroxyl radicals. Many metabolic processes, e.g., mitochondrial electron transport chain and distinct enzymes, produce free radicals, which according to the theory, can potentially damage cellular lipids, proteins and DNA. Especially with aging, this process can ultimately lead to organismal deterioration. There is solid evidence to support this theory, e.g., (i) the level of oxidative damage increases with aging in proteins, lipids and DNA, (ii) there is an extensive antioxidant system, the deficiencies of which increase oxidative damage in cells, (iii) an increased metabolic rate promotes ROS production, consistent with the rate-of-living theory [16], and (iv) mitochondrial ROS production gradually increases with aging, simultaneously with a decline in energy production capacity and increased mtDNA damages. Many review articles have been written on the role of oxidative stress in the aging process and age-related diseases [17–20]. However, all of these experimental observations are correlations rather than causal connections.

During the last decade, an increasing number of studies have appeared that have questioned the free radical theory of the aging process. Conflicting results were reported in experiments on *C. elegans* but also on rodents, which clearly revealed that ROS production does not control the aging process. Hekimi *et al.* [21] and Ristow and Schmeisser [22] have recently reviewed in detail these observations. For instance, there are long-lived species and genetic mutants displaying a high level of oxidative damage and chronic oxidative stress [23–25]. The naked mole-rat, the longest-lived rodent with a lifespan over 30 years, is a striking example refuting the age-related oxidative degeneration theory. Andziak *et al.* [23] demonstrated that naked mole-rats displayed lower antioxidant capacity, elevated lipid peroxide concentrations and significantly higher levels of oxidative damage to lipids, proteins and DNA than physiologically age-matched mice did. It seems that naked-mole rats can tolerate oxidative stress and damage better than their short-lived counterparts via mechanisms currently unknown [26]. These rats are also protected against tumorigenesis and show a striking decline in the appearance of senescent phenotype although they express clearly elevated levels of ROS and oxidative damages [27]. In addition, there are studies indicating that the increase in oxidative stress, e.g., by knocking out antioxidants, can extend the lifespan of *C. elegans* [25,28]. Moreover, superoxide dismutase, an enzyme, which is indispensable for the detoxification of superoxide radicals, is not obligatory to achieve a normal lifespan of *C. elegans* [29]. On the other hand, somatic mutations in mtDNA induce a premature aging phenotype in mice but do not affect ROS production or increase oxidative stress [30]. Sanz *et al.* [31] also conducted many experiments demonstrating that mitochondrial ROS production was not crucial for the control of lifespan in *Drosophila.* In this respect, it is no surprise that many clinical dietary antioxidant trials have not achieved any consistent health benefits but occasionally have even increased the incidence of cancers [32,33].

**Figure 1:**
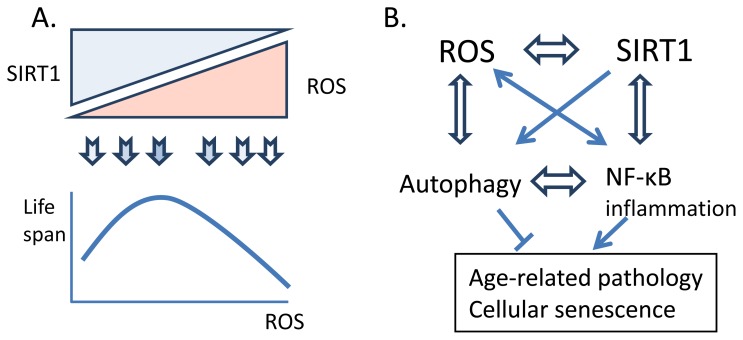
A schematic figure depicting the crosstalk between ROS production and SIRT1 activity in the regulation of age-related pathology. (**A**) The concentration-dependent regulation of the lifespan by ROS. The upper part shows the hypothetical balance between the SIRT1 activity and the presence of ROS in the control of age-related changes. There seems to be an optimal level of cellular ROS production, which confers the most favorable benefits on the healthspan and consequently extends the lifespan. (**B**) The signaling interplay between the ROS production and the SIRT1 activity, which controls the autophagy and the NF-κB signaling and consequently, induces age-related pathology and cellular senescence. Double-edged arrows indicate context-dependent interactions, not specific activation or inhibition.

Recent studies have demonstrated that reactive oxygen species are important cellular signaling molecules, similarly as reactive nitrogen species [34–36]. In this context, it is important to note that an excessive supply of synthetic antioxidants can induce antioxidative stress and the redox imbalance can lead to harmful effects [33]. The cysteine residues in proteins are the most sensitive targets for the ROS-induced oxidation. Hydrogen peroxide (H_2_O_2_), the stable form of ROS, is a potent signaling molecule inhibiting many protein phosphatases, e.g., PTEN and PP2a, and thus stimulating the growth factor pathways, e.g., insulin/PI3K/AKT signaling [35–37]. Salmeen and Barford [37] have described the chemical reactions in the redox regulation of the nucleophilic catalytic cysteines of protein and lipid phosphatases. On the other hand, ROS can activate some protein kinases, e.g., AMPK, ASK1 and Src, and also stimulate several transcription factors including HIF-α, NF-κB and NRF2 [34–36]. However, the redox balance can be restored by antioxidants, e.g., glutathione reductase and thioredoxin systems which implies that ROS have a crucial role in the regulation of signaling pathways. These examples clearly indicate that ROS perform fundamental cell survival functions by controlling cell signaling pathways and activating adaptive gene expression mechanisms whereas higher doses of ROS can directly damage proteins, lipids and DNA, as proposed by Hamanaka and Chandel [34]. Considering all these facts, the free radical theory of aging seems over-simplistic, and many researchers have refined the theory. For instance, Hekimi *et al.* [21] have proposed that there is a gradual ROS response hypothesis, which could explain the role of ROS in age-related diseases. In conclusion, it seems that an optimal level of ROS is required for a successful aging process (Figure 1). However, the basic mechanism of aging might be ROS-independent although the excessive presence of ROS with aging seems to enhance age-related degeneration and expose the individual to diseases.

### 2.2. Sirtuins as an Elixir of Longevity

Sirtuins, seven isoforms of SIRT1-SIRT7 proteins, are mammalian homologs of yeast Sir2, a silent information regulator 2, which is a class III protein deacetylase [12,38]. In 1999, Kaeberlein *et al.* [39] observed that Sir2 overexpression extended the lifespan of budding yeast. It was predicted that Sir2 could be a universal longevity factor although the aging of yeast is caused by the nucleolar fragmentation linked to the appearance of extrachromosomal rDNA circles which are not present in mammalian aging [40]. Soon after this original discovery in yeasts, it was demonstrated that the overexpression of Sir2 homologs also extended the lifespan in *C. elegans* and *Drosophila* [41,42]. Moreover, Howitz *et al.* [43] revealed that resveratrol, a polyphenol present in red wine, could activate Sir2 and prolonged the lifespan of the yeast. After that observation, resveratrol was celebrated as an elixir of longevity. Resveratrol was also claimed to extend the lifespan of *C. elegans* and *Drosophila* [44,45] but not that of wild-type mice although it improved their healthspan [46].

The recent progress on the Sir2 research has not been so spectacular. Burnett *et al.* [47] re-examined the earlier studies on the effect of Sir2 overexpression on the lifespan of *C. elegans* and *Drosophila*. Surprisingly, after the standardization of the genetic background and the use of appropriate controls, they could not find any significant lifespan extension achieved by Sir2 overexpression. They also observed that *Drosophila* Sir2 was not involved in the lifespan extension induced by dietary restriction. Currently, this topic is still a matter of fierce debate. In addition, Fabrizio *et al.* [48] conducted experiments with yeast and revealed that Sir2 could extend only the replicative lifespan but not the chronological lifespan. Recently, Park *et al.* [49] demonstrated that resveratrol was a nonselective inhibitor of cAMP-degrading phosphodiesterases including PDE1, PDE3 and PDE4. Thus, resveratrol upregulated cellular cAMP levels which increased intracellular Ca^2+^ concentration via the activation of the Epac1 pathway. Consequently, Ca^2+^ stimulated AMPK and increased cellular NAD^+^ levels. NAD^+^ is known to be an activator of SIRT1 and thus resveratrol could indirectly stimulate not only SIRT1 but also many other signaling pathways, which could affect the healthspan and lifespan. They also revealed that rolipram, an inhibitor of PDE4, conferred similar metabolic benefits as resveratrol, *i.e.*, improved mitochondrial function and protected against diet-induced obesity in mice. Moreover, Gerhart-Hines *et al.* [50] observed that the cAMP/PKA pathway phosphorylated directly the Ser434 on the SIRT1 catalytic domain, which stimulated its activity without evoking any changes in the NAD^+^ level. They also described some health benefits associated with SIRT1 activation, e.g., stimulation of fatty acid oxidation. In conclusion, the studies concerning the role of Sir2/SIRT1 in the lifespan extension are currently controversial; many of the putative beneficial effects can be linked to the capacity of SIRT1 to improve the healthspan. It is also probable that in mammals, the age-related observations are attributable to SIRT6, instead of SIRT1. Recently, Kanfi *et al.* [51] demonstrated that the overexpression of SIRT6 could increase the lifespan of male mice. It is known that SIRT6 is involved in chromatin regulation and the genome maintenance [52]. Moreover, the deletion of *Sirt6* gene induced a premature aging phenotype in mice [53].

There is substantial evidence showing that SIRT1 is a crucial regulator of mammalian energy metabolism as well as of many survival functions [12,54,55]. SIRT1 controls mitochondrial biogenesis via PGC-1α and subsequently the oxidation of energy metabolic substrates. Moreover, NAD^+^ is a potent activator of SIRT1, which makes SIRT1 to be a sensor of metabolic homeostasis. There is also an abundant literature indicating that the functional dysregulation of SIRT1 is associated with many age-related diseases, e.g., metabolic syndrome, cardiovascular and neurodegenerative diseases, and cancer [12,54,56]. Currently, it seems that SIRT1 is not the fountain of youth, as proposed ten years ago, but a major regulator of metabolic and survival functions and, in that way, an important contributor to the maintenance of a healthy aging process. Thus, SIRT1 represents a significant therapeutic target in the drug discovery programs concentrating on age-related diseases [57].

## 3. Signaling Crosstalk between ROS and SIRT1

### 3.1. SIRT1 Controls ROS Level

Alcendor *et al.* [58] revealed that there was an extensive crosstalk between the SIRT1 expression and the level of oxidative stress in mouse cardiac muscle. Using the overexpression technique, they were able to demonstrate that the moderate overexpression of SIRT1 could protect against oxidative stress by inducing the expression of catalase, a major antioxidant, and it also attenuated the appearance of age-related cardiomyopathy involving hypertrophy. In contrast, the high level of SIRT1 expression (12.5-fold increase) clearly increased oxidative stress and evoked pathological changes in the heart. It should be noted that they did not record whether SIRT1 was functionally active or inhibited by excessive ROS, which could provoke cardiac pathology (Section 3.2.). Overexpressed proteins are commonly prone to aggregate, a factor that could stimulate oxidative stress. On the other hand, Alcendor *et al.* [58] confirmed that the increase in catalase expression was induced by the FoxO1 transcription factor. There is a substantial literature demonstrating that SIRT1 can deacetylate the FoxO factors, *i.e.*, FoxO1, FoxO3a and FoxO4, and subsequently stimulate the expression of antioxidants, e.g., catalase, MnSOD and Trx, and via an auto-feedback loop also potentiate SIRT1 expression [59–63]. The SIRT1/FoxO axis is an evolutionarily well conserved survival pathway that regulates cellular responses to both metabolic changes and many stress insults including oxidative stress. FoxO1 and FoxO3 can also promote autophagy [61], a process which declines with aging and is disturbed in several age-related diseases (Section 4.).

SIRT1 regulates immune responses via NF-κB signaling and in that way also controls the ROS production (Section 4.2.). Yeung *et al.* [64] revealed that SIRT1 could inhibit the transactivation capacity of the NF-κB complex by deacetylating the Lys310 residue of RelA/p65 component. Subsequently, many studies have demonstrated that SIRT1 is a potent intracellular inhibitor of oxidative stress and inflammatory responses [65–67]. In addition to mitochondria, NADPH oxidases, including an evolutionarily conserved Nox family [68], produce superoxide radicals. The mammalian Nox1-4 enzymes are commonly expressed in mammalian tissues, especially in professional phagocytes. NF-κB signaling, a major immune defense system, is a potent inducer of the expression of NADPH oxidase components, e.g., gp91*^phox^* and p22*^phox^* [69–71]. Moreover, NF-κB signaling also transactivates the iNOS expression and thus increases the production of reactive nitrogen radicals [72,73]. However, NF-κB signaling can also induce the expression of several antioxidants, e.g., MnSOD, Cu,Zn-SOD and Trx1 [74], which means that in the crosstalk with NF-κB system, SIRT1 can not only repress the ROS production but also reduce the antioxidant defense. In the Section 4.2., we will discuss this topic in greater detail with respect to aging and age-related diseases.

SIRT1 can also inhibit some other transcription factors, which are involved in the regulation of cellular redox balance. Kawai *et al.* [75] demonstrated that SIRT1 inhibited the transactivation capacity of NRF2 by deacetylating the Lys588 and Lys591 residues, which subsequently suppressed the binding of NRF2 to its cognate DNA response element ARE (antioxidant response element). The ARE site is an important *cis*-element in several antioxidant genes e.g., driving the expression of glutathione peroxidase 2, peroxiredoxin 4 and thioredoxin reductase [76–78]. Kawai *et al.* [75] revealed that the SIRT1-mediated deacetylation of NRF2 protein terminated the transcription of antioxidant genes and consequently, NRF2 was translocated out of the nuclei into the cytoplasm. They also reported that an exposure to resveratrol, a putative SIRT1 activator, inhibited the nuclear accumulation of NRF2 after an oxidative insult. These studies indicated that the overexpression of SIRT1 could disturb cellular redox balance by repressing the NRF2-induced antioxidant defense. On the other hand, oxidative stress can activate the NRF2 signaling through the inactivation of Keap1, an inhibitor of NRF2 [79]. Keap1 is degraded by autophagy and thus autophagy can control the cellular redox status [80] (Section 4.1.). Moreover, the decline in autophagic flux increases the protein level of p62, a selective autophagy receptor, which consequently dismantles the Keap1/NRF2 complex and thus stimulates the NRF2-mediated transcription, which potentiates the antioxidant defense [81]. In addition, the NRF2 signaling can enhance the expression of p62 and subsequently augment autophagy since the promoter of p62 contains the ARE sequence [82]. These studies indicate that there is a complex interplay between SIRT1 and the Keap1/NRF2 signaling in the control of the cellular redox homeostasis and autophagy (Section 4.1.).

Mitochondria are a major source of ROS production in most of the mammalian tissues. The mitochondrial electron transport chain, especially complexes I and III, generates superoxide radicals, the intensity of production being dependent on the level of oxygen and respiratory activity [83]. PGC-1α is a transcriptional co-activator, which regulates mitochondrial biogenesis through the control of many transcription factors involved in the expression of mitochondrial proteins [84,85]. Certain signaling pathways can regulate the expression level of PGC-1α protein; however, more importantly, some post-translational modifications control the transactivation capacity of PGC-1α protein. SIRT1 is one of these factors, which affect mitochondrial respiration, and thus ROS production via the modulation of PGC-1α activity. Currently, there are controversial results concerning the role of SIRT1 in the regulation of mitochondrial respiration through the deacetylation of PGC-1α. Nemoto *et al.* [86] demonstrated that SIRT1 physically interacted with PGC-1α protein and induced its deacetylation. They reported that this deacetylation reaction inhibited the transcriptional activity of PGC-1α protein and, consistently, the overexpression of SIRT1 reduced oxygen consumption in PC12 cells. Several subsequent studies have confirmed that SIRT1 could deacetylate PGC-1α factor although the effects have been somewhat inconclusive. Rodgers *et al.* [87] observed that the deacetylation of PGC-1α stimulated the expression of gluconeogenic genes but did not affect the regulation on the mitochondrial genes in the rat liver. However, there are studies indicating that SIRT1 could activate the PGC-1α-mediated expression of mitochondrial respiratory genes and thus enhance respiration, particularly in skeletal muscles [10,50,88]. Recently, Philp *et al.* [89] demonstrated with transgenic mice lacking the SIRT1 deacetylase activity that SIRT1 was involved neither in the endurance exercise-induced deacetylation of PGC-1α nor in the subsequent mitochondrial biogenesis. Moreover, Amat *et al.* [90] revealed that SIRT1 stimulated the transcription of PGC-1α gene together with MyoD in myogenic cells. It seems that SIRT1 can regulate the PGC-1α-mediated mitochondrial respiration and thus the ROS production via several distinct mechanisms in a context-dependent manner.

There is a substantial literature indicating that overwhelming ROS production during intensive aerobic respiration can be uncoupled by respiratory uncoupling proteins (UCP). UCP2 and UCP3, two tissue-specific uncoupling proteins, can attenuate the mitochondrial ROS production and thus protect cells against oxidative damage [91,92]. Andrews and Horwath [93] demonstrated that increased expression of UCP2 could reduce the ROS production and oxidative stress in the tissues of mice and consequently extend the lifespan of the animals. Vidal-Puig *et al.* [94] also observed that the knockout of the *UCP*3 gene increased the mitochondrial ROS production in mouse skeletal muscle. Moreover, it does seem that there is a negative feedback control of ROS production in the mitochondria since an increased level of ROS can activate UCP2 and UCP3 proteins [92]. These observations agree with the hypothesis of "mitochondrial uncoupling-to-survive" theory of aging. In this respect, there are several interesting studies indicating that SIRT1 can repress the expression of UCP2 and UCP3 [95,96]. Bordone *et al.* [95] demonstrated that SIRT1 could bind directly to the promoter region of *UCP2* gene and inhibit UCP2 expression in pancreatic β cells. Subsequently, while confirming this observation, they also revealed that the UCP2 levels were increased in the pancreas of SIRT1 knockout mice and in the knockdown β cells. The down-regulation of UCP2 expression provoked glucose-stimulated insulin secretion. Moreover, Amat *et al.* [96] observed that treatment with glucocorticoids induced the expression of UCP3 in skeletal muscle where it was preferentially expressed. They also demonstrated that SIRT1 was a potent repressor of glucocorticoid-induced UCP3 expression due to its ability to prevent the interaction between the glucocorticoid receptor and p300 histone acetyltransferase. In conclusion, it seems that SIRT1 can enhance mitochondrial ROS production by suppressing UCP expression, although currently it is not known whether uncoupling is the major function of UCPs in mitochondria. For instance, UCP2 has also been associated with the glucose and lipid metabolism in mitochondria [97].

### 3.2. ROS Control SIRT1 Activity

There is accumulating evidence indicating that an increased ROS level can directly or indirectly control the activity of SIRT1 enzyme. The excessive presence of ROS, e.g., during aging and in several age-related diseases, complicates the estimation of the functional role of SIRT1 in these conditions. Kamata *et al.* [98] demonstrated that ROS induced a sustained activation of JNK1 by inhibiting JNK phosphatases, e.g., MKP-1, MKP-3, and MKP-5. ROS could also activate JNK1 by stimulating the redox-regulated ASK1 kinase [99]. Recently, Nasrin *et al.* [100] revealed that oxidative stress triggered the interaction of JNK1 with SIRT1 and consequently, JNK1 phosphorylated SIRT1 on Ser27, Ser47 and Thr530 residues. This modification increased the activity of SIRT1 and induced its translocation into the nuclei. Interestingly, the activation of SIRT1 by JNK1 specifically deacetylated histone H3 but not p53, a major target of SIRT1. This finding implies that ROS can regulate directly gene expression via the JNK1-SIRT1 link, in addition to their effects mediated through signaling pathways. On the other hand, Gao *et al.* [101] demonstrated that the persistent activation of JNK1 achieved by treatment with insulin or glucose triggered the ubiquitination of SIRT1 protein and subsequently its degradation via proteasomes in mouse liver. The depletion of SIRT1 promoted the appearance of hepatic steatosis in mice. However, the ROS-activated JNK pathway could also generate a tolerance to oxidative stress and consequently prolong the lifespan in *Drosophila* [102]. This may be associated with the JNK-mediated activation of FoxO factors and the ability of JNK to inhibit insulin signaling [103]. Both of these effects are evolutionarily conserved longevity mechanisms which can also protect against age-related metabolic diseases.

In addition to modulating the JNK signaling, ROS also control the activation of AMPK, a major regulator of metabolic homeostasis [104]. Remarkably, AMPK can reciprocally activate SIRT1 by increasing cellular NAD^+^ synthesis [105]. AMPK is a redox-sensing enzyme, and both oxygen and nitrogen radicals, *i.e.*, H_2_O_2_ and NO, can oxidize distinct cysteine residues of AMPK subunits producing *S*-hydroxylated and *S*-nitrosylated thiols which subsequently react with reduced glutathione (GSH) generating S-glutathionylated derivatives of AMPK components [104]. Zmijevski *et al.* [106] demonstrated that H_2_O_2_ triggered the *S*-glutathionylation of both AMPKα and AMPKβ subunits, a modification which increased the activity of the AMPK complex. In particular, the Cys299 and Cys304 amino acids in the AMPKα subunit were sensitive towards cysteine oxidation. Furthermore, Mungai *et al.* [107] revealed that the hypoxia-induced ROS production triggered Ca^2+^ release from the endoplasmic reticulum stimulating CaMKKβ, which subsequently activated AMPK signaling. In hypoxia, AMPK is activated by mitochondria-derived ROS rather than increased AMP/ATP ratio [108]. NO can also activate AMPK through the stimulation of CaMKKβ in vascular endothelial cells [109]. These studies indicate that AMPK is a sensitive redox target as well as a metabolic gauge. It is well known that AMPK signaling is linked downstream to an integrated signaling network, including also SIRT1, which controls both the aging process and age-related diseases [110].

Oxidative stress can inhibit the SIRT1 mRNA level by inducing the expression of microRNAs. Yamakuchi *et al.* [111] revealed that miR-34a could bind to 3′UTR of SIRT1 mRNA and inhibit the SIRT1 expression. Consequently, miR-34a increased the acetylation of p53, a major deacetylation target of SIRT1, and it could induce a cancer cell apoptosis. Interestingly, there appears to be a positive feedback loop since p53 can stimulate the transcription of miR-34a, which blocks the expression of SIRT1, an inhibitor of p53. MiR-34a has several target mRNAs and it controls the cell cycle, apoptosis and metabolism [112]. MiR-34a also targets the mRNAs of some antioxidants, e.g., superoxide dismutase 2 and thioredoxin reductase 2 [113]. Recently, Li *et al.* [114] reported that the expression of miR-34a increased in rat liver with aging, and accordingly the expression of SIRT1 decreased. In endothelial cells, overexpression of miR34a provoked cellular senescence [115]. There is a substantial literature indicating that oxidative stress can either stimulate or suppress the p53-driven transcription [116,117], and thus it may control SIRT1 levels via the expression of miR-34a. Moreover, Guo *et al.* [118] demonstrated that oxidative stress directly activated ATM protein kinase, an upstream activator of p53, by inducing the oxidation of specific cysteine residues in ATM protein. However, it is clear that p53 protein itself can also undergo several context-dependent, redox-related modifications, e.g., oxidation of cysteines and nitration of tyrosines, which affect its functional properties [117,119].

There are studies indicating that the cysteine residues of SIRT1 are vulnerable to oxidation in conditions of oxidative stress and that this affects both the activity of SIRT1 and its degradation in the proteasomes [120–122]. Although SIRT1 inhibits NF-κB signaling and suppresses inflammation (Section 3.1.), the kind of chronic oxidative stress encountered in inflammatory conditions clearly down-regulates the expression and activity of SIRT1 [123]. Interestingly, Cai *et al.* [122] observed that the chronic oral administration of the pro-oxidative advanced glycation endproduts (AGE) strongly reduced the levels of SIRT1 protein in the mouse white adipose tissue, liver and skeletal muscle. This change was associated with enhanced adiposity and insulin resistance, which are typical features of type 2 diabetes. Caito *et al.* [121] reported that oxidant/aldehyde stress increased the appearance of carbonylation and alkylation of SIRT1 cysteine groups and induced the concomitant degradation of SIRT1 protein in the proteasomes. They also observed that the redox status, especially the level of intracellular thiols, affected the loss of SIRT1 during exposure to environmental stress. Oxidative stress can also induce a transient S-glutathionylation of SIRT1 [124]. Kornberg *et al.* [125] demonstrated that SIRT1 could also be nitrosylated via the GAPDH-mediated transnitrosylation reaction, which clearly reduced the activity of SIRT1. Moreover, oxidative stress can decrease the level of NAD^+^ and thus inhibit SIRT1 activity [120]. These studies clearly indicate that SIRT1 enzyme itself can be the target of oxidative modifications and become inhibited during oxidative stress.

Caito *et al.* [121] reported that the exposure of lung epithelial cells to oxidative stress by H_2_O_2_ and cigarette smoke extract induced the translocation of SIRT1 from the nuclei to cytoplasm. More recently, Tong *et al.* [126] observed that ischemic stress also provoked the translocation of nuclear SIRT1 to the cytoplasm in cardiomyocytes. They also revealed that SIRT1 was sumoylated in the nuclei of cardiomyocytes under normal physiological conditions, whereas ischemic stress induced a desumoylation and translocation of SIRT1 into the cytoplasm. Interestingly, this process was dependent on the age of animals, *i.e.*, nuclear-to-cytoplasmic shuttling was significantly increased in aged mice compared to their younger counterparts, which did not exhibit any cytoplasmic translocation. Moreover, ischemic insult strongly increased the cardiac NAD^+^ level in young mice but not in old animals enhancing the activity of SIRT1. Yang *et al.* [127] demonstrated that the sumoylation of SIRT1 at Lys734 increased its deacetylase activity whereas desumoylation by SENP1 had an opposite effect. They also observed that oxidative stress evoked by UV-radiation and H_2_O_2_ promoted the interaction of SIRT1 with SENP1 leading to the desumoylation and inhibition of SIRT1 activity. These studies indicate that sumoylation can enhance the activity of SIRT1, which consequently prevents apoptotic cell death. In contrast, oxidative stress stimulates desumoylation and exposes cardiomyocytes to apoptosis. Currently, it is not known whether this mechanism is present in any other tissues in addition to heart and lung.

## 4. Age-Related Effects of the Crosstalk between ROS and SIRT1

There is a growing literature revealing that autophagy is impaired with aging and consequently, a low-grade inflammatory phenotype prevails in aging tissues [5,8,128–131]. Since SIRT1 and oxidative stress are important regulators of both autophagy and inflammation (see below), it seems that the context-dependent control of the balance between SIRT1 and ROS may play a crucial role in the appearance of these hallmarks related to both the aging process and several age-related diseases. Currently, no distinct mechanism is known through which autophagy would decline during aging or whether this process actually controls the aging process or not. However, it seems that the deficiency in autophagy could enhance inflammatory responses in tissues and induce a state called inflammaging [8].

### 4.1. Autophagy

Autophagy is an evolutionarily conserved cellular housekeeping mechanism which removes aberrant cellular constituents or supplies energy by self-eating during starvation [132,133]. Thus, it is not surprising that there are studies indicating that SIRT1 is a potent inducer of autophagy [11,134,135]. In their seminal work, Lee *et al.* [134] demonstrated that SIRT1 could directly affect autophagic flux by controlling the Atg8 (LC3) and Atg12 conjugation systems. They revealed that SIRT1 deacetylated Atg5, Atg7, and Atg8 proteins to stimulate autophagosome formation, both *in vitro* and *in vivo*. Embryonic fibroblasts from these mice could not perform autophagic induction in response to starvation. These workers also observed that the absence of SIRT1 induced the accumulation of damaged organelles, e.g., abnormally shaped mitochondria, and disturbances in energy homeostasis in neonatal SIRT1 knockout mice. Moreover, the SIRT1^−/−^ mice resembled those of Atg5^−/−^ in many respects, *i.e.*, both strains displayed disturbances in energy metabolism and suffered early perinatal mortality.

In addition to the direct control of autophagosome formation, SIRT1 can also regulate autophagy indirectly via the FoxO signaling. Hariharan *et al.* [136] demonstrated that SIRT1 deacetylated FoxO1 during glucose starvation, a condition that stimulates autophagy in cardiac myocytes. Studies elucidating the mechanism, they revealed that deacetylated FoxO1 could stimulate the expression of Rab7 protein. Rab7, a small GTP-binding protein, is a crucial factor in the maturation of autophagosomes and endosomes, inducing their fusion with lysosomes [137]. Hariharan *et al.* [136] also reported that the knockdown of Rab7 inhibited the FoxO1-induced autophagy, highlighting the critical role of Rab7 in the SIRT1-induced autophagy. Moreover, they demonstrated that the interaction between SIRT1 and FoxO1 was also required *in vivo* in the starvation-induced autophagy in murine heart. Brunet *et al.* [59] observed that SIRT1 could deacetylate FoxO3 in response to oxidative stress and thus improve the cellular stress resistance. Later studies have revealed that FoxO3 stimulated the expression of autophagy-related proteins, e.g., LC3 and Bnip3, and subsequently induced autophagic protein degradation in skeletal muscle during fasting [138]. Although there are several studies indicating that the overexpression of SIRT1 can have beneficial effects in the myocardium, these being probably mediated by autophagy [136,139] as in neurons [140], there is clear evidence that the long-lasting overexpression of SIRT1 can have deleterious effects on cardiac muscle, e.g., impairing mitochondrial biogenesis and morphology as well as reducing the functional capacity of heart [58,141,142]. A persistent, non-physiological overexpression of SIRT1 could expose cells to excessive autophagy, even autophagic cell death (Figure 1).

There is a mounting literature indicating that ROS are potent inducers of autophagy, at least under experimental conditions [143–145]. The ability of ROS to initiate autophagy seems somewhat contradictory in the light of the increased oxidative stress and decreased autophagy present during aging. Superoxide radicals produced by mitochondria, NADPH oxidases and xanthine oxidases appear to be the most crucial stimulators, which trigger the autophagic process [143,145,146]. Currently, there are several mechanisms that are known to be involved in mediating ROS-induced autophagy in experimental contexts but what is their role in autophagy in normal and pathological conditions still remains to be determined. Scherz-Shouval *et al.* [147] demonstrated that ROS oxidized the Cys77 and Cys81 residues of Atg4 protein. Atg4 is a cysteine protease, which cleaves Atg8 (LC3) and thus prevents the lipidation required for autophagosome formation. Several antioxidants were able to abolish the lipidation of LC3 and subsequent autophagy. They also reported that the autophagy induced by starvation was dependent on the ROS-mediated LC3 inactivation.

The presence of ROS can also control autophagy via the activation of different protein kinases, e.g., JNK1 and AMPK (Section 3.2.). Wei *et al.* [148] demonstrated that JNK1 phosphorylated the Bcl-2 protein, which induced the dissociation of Bcl-2 from Beclin 1, and consequently activated autophagy. Beclin 1 is a crucial stimulator of autophagosome formation. Interestingly, these workers observed that starvation-induced autophagy was dependent on the JNK1 activation, probably stimulated by ROS (see below). Park *et al.* [149] revealed that JNK1 activation could upregulate Beclin 1 expression and induce Bcl-2 and p53 phosphorylation, leading to autophagic cell death. JNK1 can also trigger autophagy by increasing the expression of Sestrin 2 [150]. However, the autophagic responses induced by the JNK pathway seem to be context-dependent in different tissues. Xu *et al.* [151] established a mouse model conditionally deficient of *Jnk* gene in neurons. Surprisingly, they observed that the neurons of these mice displayed an increased autophagy, which was mediated via the FoxO/Bnip3/Beclin 1 pathway. Moreover, Sarkar *et al.* [152] demonstrated that nitric oxide (NO), in contrast to ROS, inhibited JNK1 by *S*-nitrosylation and subsequently suppressed autophagy in rat primary neurons. However, Wang *et al.* [102] demonstrated that JNK signaling could induce a gene expression program, which enhanced tolerance to oxidative stress and extended the lifespan of *Drosophila*. These contradictory results imply that there are complex interactions, probably tissue and species-specific, involved in the crosstalk between the redox status and JNK signaling pathway.

In addition to their effects on JNK signaling, ROS can also activate autophagy by stimulating AMPK [153,154] or p38MAPK [155,156]. Alexander *et al.* [153] demonstrated that oxidative stress activated ATM, which subsequently stimulated AMPK via LKB1. They observed that AMPK activated TSC2, an inhibitor of mTOR and thus triggered autophagy. AMPK can also induce autophagy by stimulating ULK1 [157] or FoxO3a [158]. Interestingly, there are several upstream effectors which activate AMPK in oxidative stress, e.g., (i) ROS-mediated oxidation and S-glutathionylation of AMPK (Section 3.2.), (ii) ROS-induced leakage of Ca^2+^ and subsequent activation of CaMKKβ (Section 3.2.) and (iii) ATM, a cellular damage sensor, particularly that of genotoxic stress [159]. It seems possible that by activating the AMPK kinase, ROS can control an integrated signaling network linking AMPK to several longevity factors including p53, CRTC-1, FoxOs, NF-κB, NRF2 and Sestrins [110]. Similar to the way that ROS can exert dual responses, autophagy has also its beneficial health effects and detrimental outcomes in autophagic cell death. In order to maintain cellular housekeeping, autophagy can control any excessive ROS production by eliminating dysfunctional mitochondria through mitophagy [160]. However, the age-related decline in autophagy disturbs the cellular quality control by enhancing the appearance of dysfunctional, ROS-producing mitochondria, which trigger inflammatory responses by stimulating inflammasomes [9].

### 4.2. NF-κB and Inflammation

The NF-κB signaling is a crucial regulator of immune defense system and an inducer of inflammatory responses [161]. The NF-κB system is also involved in many housekeeping and survival functions during cellular stress e.g., by controlling apoptosis, proliferation and energy metabolism [162–164]. Both SIRT1 and oxidative stress are known to be able to regulate NF-κB signaling and in that way are crucially involved in the maintenance of cellular homeostasis [64,74] (Figure 1). There are several studies demonstrating that NF-κB signaling is activated during aging [165–168]. A low-grade inflammation is a characteristic aging phenotype at both the tissue and organismal levels as detected by different technical approaches, e.g., by microarrays [129,169]. As described earlier (Section 3.1.), SIRT1 is a potent inhibitor of NF-κB signaling and thus it suppresses inflammation [64,65]. Many downstream targets of SIRT1 also repress inflammatory responses, e.g., AMPK [67] and FoxO factors [170], by inhibiting the NF-κB signaling. Interestingly, Gillum *et al.* [171] demonstrated that the expression level of SIRT1 controlled the recruitment of macrophages into adipose tissue during chronic feeding of a high-fat diet. The reduction of SIRT1 expression in mouse adipose tissue decreased histone H3K9 deacetylation and activated NF-κB signaling. Consequently, increased cytokine expression triggered macrophage infiltration. On the other hand, the overexpression of SIRT1 prevented macrophage recruitment and inflammation during consumption of the high-fat diet [171]. This study indicated that SIRT1 could also regulate the expression of inflammatory genes at chromatin level, in addition to direct repression of NF-κB signaling. Recently, Liu *et al.* [172] demonstrated that LPS treatment induced the binding of SIRT1 to the promoters of TNF-α and IL-1β and the deacetylation of RelA/p65 and histone H4K16 consequently suppressed the expression of these cytokines. Subsequently, SIRT1 formed a mature repressor complex with RelB to these promoters and thus generated endotoxin tolerance through the epigenetic reprogramming in THP-1 cells.

The crosstalk between oxidative stress and inflammation is a complex process and there is an abundant literature focusing on age-related degenerative and inflammatory diseases [173,174]. Briefly, oxidative stress and excessive presence of ROS are able to stimulate the NF-κB system and thus generate inflammatory responses [175]. However, there are several activation mechanisms and it seems to be a cell-type specific process. Moreover, there are reports that ROS can stimulate inflammation via the activation of inflammasomes and the production of IL-1β and IL-18 cytokines, which subsequently trigger inflammatory responses [6,9]. Currently, the precise mechanism still needs to be verified although the TXNIP-mediated activation is the most probable pathway [176]. There is a plethora of articles indicating that antioxidants, e.g., dietary polyphenols, can inhibit inflammation. It is well known that many polyphenols, such as terpenoids, are able to inhibit NF-κB signaling and thus repress inflammation [177,178]. However, clinical experiments have failed to demonstrate any convincing therapeutic potency [32]. As described earlier, there are copious mutual interactions and a delicate balance between SIRT1 and ROS signaling which provoke context-dependent responses to autophagic flux and inflammation. Moreover, there is a reciprocal control network between the IKK-NF-κB signaling and autophagy [179], which regulates inflammatory responses in a context-dependent manner. Currently, it seems that aging adds its own distinct flavoring to this balance and generates the hallmarks of aging process, which are probably context-dependently potentiated in age-related degenerative diseases.

### 4.3. SIRT1, ROS and Insulin/IGF-1 Paradox of Aging

The insulin/IGF-1 paradox of aging was discovered in the studies on the long-lived mutants of *Caenorhabditis elegans* [180]. Several lines of experiments revealed that the loss-of-function mutations disturbing the DAF-2 pathway extended the lifespan of *C. elegans* and could induce the dauer phenotype [180,181]. The DAF-2 pathway is an ortholog to the mammalian signaling pathway stimulated by insulin/IGF-1 receptors through the PI-3K/AKT signaling. Many studies have demonstrated that the inhibition of this evolutionarily conserved signaling pathway can extend the organismal lifespan, even in mammals [182,183]. The DAF-16/FoxO transcription factors are the crucial downstream targets of the insulin/IGF-1 cascade, which suppresses the function of FoxO factors. The insulin/IGF-1 signaling is a somatotropic pathway, which also controls protein synthesis and many metabolic actions. A deficient activation of this pathway may lead to dwarfism. There are many models of dwarf mice, which live 20% to 70% longer than their wild type counterparts [184]. As described in Sections 3.1. and 4.1., FoxO factors stimulate the expression of several antioxidants and SIRT1 as well as many autophagy proteins. These responses increase stress resistance, which is a hallmark of long-lived organisms [185]. Moreover, it is known that the insulin/IGF-1 pathway can enhance inflammation via the activation of the AKT/IKK/NF-κB signaling. Therefore, the repression of insulin/IGF-1 signaling could also reduce the level of inflammatory responses [186,187]. On the other hand, increased IKKβ activation can induce insulin resistance, e.g., in liver, by increasing systemic level of inflammatory mediators [188].

Growth hormone stimulates the synthesis of IGF-1 in the liver but several tissues can also produce tissue-specific IGF-1 isoforms for paracrine and autocrine actions. For instance, the cardiac and skeletal muscles and the brain can synthesize IGF-1 peptide isoforms for local regulation [189–191]. Recent studies have indicated that the IGF-1 isoforms expressed by liver or locally in tissues have clearly different functional properties. Vinciguerra *et al.* [191] demonstrated that the local, mIGF-1, rather than the systemic IGF-1 isoform increased SIRT1 expression and its catalytic activity in mouse cardiomyocytes. Several studies indicate that mIGF-1 can protect the heart against oxidative stress and the angiotensin II-induced hypertrophy of cardiomyocytes [191–193]. Vinciguerra *et al.* [193] confirmed that the protection against oxidative stress was dependent on the SIRT1 activity in transgenic mIGF-1 mice. They also revealed that JNK-1 activity was required for the induction and activation of SIRT1 in mouse cardiomyocytes. This observation clearly indicates that mIGF-1 and IGF-1 have distinct signaling pathways and SIRT1 is linked to the regulation of local IGF-1.

There are controversial observations on the role of SIRT1 in the neuronal survival associated with the insulin/IGF-1 signaling [189,194–196]. Li *et al.* [195] observed that the inhibition of SIRT1 increased oxidative stress resistance in the cultured rat neurons. A reduced level of oxidative stress and lipid peroxidation markers in the brains of SIRT1 knockout mice were also recorded. In addition, the inhibition of SIRT1 increased the acetylation but decreased the phosphorylation level of IRS-2 protein which is a key component in the insulin/IGF-1 signaling. This implies that SIRT1 can enhance the insulin/IGF-1 signaling. Zhang [197] detected that SIRT1 deacetylated IRS-2 protein which crucially increased the phosphorylation of IRS-2 and promoted insulin/IGF-2 signaling. It seems that SIRT1 is involved in the regulation of the insulin/IGF-1 aging paradox through the IRS-2 and FoxO signaling (Section 3.1.).

## 5. Conclusions

Accumulating evidence indicates that there is a need to refine the two present aging dogmas, *i.e.*, the free radical theory of aging and the role of SIRT1 as a major longevity factor. Recent studies have revealed that ROS, at the physiological level, are crucial signaling molecules and SIRT1 protein is an important survival factor but not the fountain of youth. Furthermore, there is a complex interplay between the regulation of cellular activity of SIRT1 and the presence of ROS. In a context-dependent manner, these factors can control reciprocally each other’s functional activities, directly or via an integrated signaling network. ROS and SIRT1 are major regulators of autophagy and inflammatory responses, both of which are disturbed in the aging process. It seems that there is an optimal balance between the level of ROS production and SIRT1 activity, which confers the most favorable benefits on the health span; consequently, maintenance of this balance can even extend the lifespan of an organism.

## References

[1] Green D.R., Galluzzi L., Kroemer G. (2011). Mitochondria and the autophagy-inflammation-cell death axis in organismal aging. Science.

[2] Koga H., Kaushik S., Cuervo A.M. (2011). Protein homeostasis and aging: the importance of exquisite quality control. Ageing Res. Rev.

[3] Baraibar M.A., Liu L., Ahmed E.K., Friguet B. (2012). Protein oxidative damage at the crossroads of cellular senescence, aging, and age-related diseases. Oxid. Med. Cell. Longev.

[4] Cui H., Kong Y., Zhang H. (2012). Oxidative stress, mitochondrial dysfunction, and aging. J. Signal. Trans.

[5] Salminen A., Kaarniranta K. (2009). Regulation of the aging process by autophagy. Trends Mol. Med.

[6] Tschopp J., Schroder K. (2010). NLRP3 inflammasome activation: The convergence of multiple signalling pathways on ROS production?. Nat. Rev. Immunol.

[7] Gross O., Thomas C.J., Guarda G., Tschopp J. (2011). The inflammasome: An integrated view. Immunol. Rev.

[8] Salminen A., Kaarniranta K., Kauppinen A. (2012). Inflammaging: Disturbed interplay between autophagy and inflammasomes. Aging (Albany NY).

[9] Salminen A., Ojala J., Kaarniranta K., Kauppinen A. (2012). Mitochondrial dysfunction and oxidative stress activate inflammasomes: Impact on the aging process and age-related diseases. Cell. Mol. Life Sci.

[10] Canto C., Auwerx J. (2009). PGC-1α, SIRT1 and AMPK, an energy sensing network that controls energy expenditure. Curr. Opin. Lipidol.

[11] Salminen A., Kaarniranta K. (2009). SIRT1: Regulation of longevity via autophagy. Cell. Signal.

[12] Haigis M.C., Sinclair D.A. (2010). Mammalian Sirtuins: Biological insights and disease relevance. Annu. Rev. Pathol. Mech. Dis.

[13] Yu J., Auwerx J. (2010). Protein deacetylation by SIRT1: An emerging key post-translational modification in metabolic regulation. Pharmacol. Res.

[14] Harman D. (1956). Aging: A theory based on free radical and radiation chemistry. J. Gerontol.

[15] Harman D. (1972). The biologic clock: The mitochondria?. J. Am. Geriatr. Soc.

[16] Hulbert A.J., Pamplona R., Buffenstein R., Buttemer W.A. (2007). Life and death: Metabolic rate, membrane composition, and life span of animals. Physiol. Rev.

[17] Berlett B.S., Stadtman E.R. (1997). Protein oxidation in aging, disease, and oxidative stress. J. Biol. Chem.

[18] Beckman K.B., Ames B.N. (1998). The free radical theory of aging matures. Physiol. Rev.

[19] Cadenas E., Davies K.J. (2000). Mitochondrial free radical generation, oxidative stress, and aging. Free Radic. Biol. Med.

[20] Dröge W (2002). Free radicals in the physiological control of cell function. Physiol. Rev..

[21] Hekimi S., Lapointe J., Wen Y. (2011). Taking a “good” look at free radicals in the aging process. Trends Cell. Biol.

[22] Ristow M., Schmeisser S. (2011). Extending life span by increasing oxidative stress. Free Radic. Biol. Med.

[23] Andziak B., O’Connor T.P., Qi W., DeWaal E.M., Pierce A., Chaudhuri A.R., van Remmen H., Buffenstein R. (2006). High oxidative damage levels in the longest-living rodent, the naked mole-rat. Aging Cell.

[24] Buffenstein R., Edrey Y.H., Yang T., Mele J. (2008). The oxidative stress theory of aging: Embattled or invincible? Insights from non-traditional model organisms. AGE.

[25] Van Raamsdonk J.M., Hekimi S. (2010). Reactive oxygen species and aging in *Caenorhabditis elegans*: Causal or casual relationship?. Antioxid. Redox Signal.

[26] Lewis K.N., Mele J., Hornsby P.J., Buffenstein R. (2012). Stress resistance in the naked mole-rat: The bare essentials—A mini-review. Gerontology.

[27] Buffenstein R. (2008). Negligible senescence in the longest living rodent, the naked mole-rat: Insights from a successfully aging species. J. Comp. Physiol. B.

[28] Van Raamsdonk J.M., Hekimi S. (2009). Deletion of the mitochondrial superoxide dismutase sod-2 extends lifespan in *Caenorhabditis elegans*. PLoS Genet.

[29] Van Raamsdonk J.M., Hekimi S. (2012). Superoxide dismutase is dispensable for normal animal lifespan. Proc. Nat. Acad. Sci. USA.

[30] Trifunovic A., Hansson A., Wredenberg A., Rovio A.T., Dufour E., Khvorostov I., Spelbrink J.N., Wibom R., Jacobs H.T., Larsson N.G. (2005). Somatic mtDNA mutations cause aging phenotypes without affecting reactive oxygen species production. Proc. Natl. Acad. Sci. USA.

[31] Sanz A., Fernandez-Ayala D.J.M., Stefanatos R.K.A., Jacobs H.T. (2010). Mitochondrial ROS production correlates with, but does not directly regulate lifespan in drosophila. Aging (Albany NY).

[32] Berger R.G., Lunkenbein S., Ströhle A., Hahn A. (2012). Antioxidants in food: Mere myth or magic medicine?. Crit. Rev. Food Sci. Nutr.

[33] Poljsak B., Milisav I. (2012). The neglected significance of “antioxidative stress”. Oxid. Med. Cell. Longev.

[34] Hamanaka R.B., Chandel N.S. (2010). Mitochondrial reactive oxygen species regulate cellular signaling and dictate biological outcomes. Trends Biochem. Sci.

[35] Gough D.R., Cotter T.G. (2011). Hydrogen peroxide: A Jekyll and Hyde signalling molecule. Cell Death Dis.

[36] Ray P.D., Huang B.W., Tsuji Y. (2012). Reactive oxygen species (ROS) homeostasis and redox regulation in cellular signaling. Cell Signal.

[37] Salmeen A., Barford D. (2005). Functions and mechanisms of redox regulation of cysteine-based phosphatases. Antioxid. Redox Signal.

[38] Blander G., Guarente L. (2004). The Sir2 family of protein deacetylases. Annu. Rev. Biochem.

[39] Kaeberlein M., McVey M., Guarente L. (1999). The SIR2/3/4 complex and SIR2 alone promote longevity in *Saccharomycetes cerevisiae* by two different mechanisms. Genes Dev.

[40] Sinclair D.A., Guarente L. (1997). Extrachromosomal rDNA circles—A cause of aging in yeast. Cell.

[41] Tissenbaum H.A., Guarente L. (2001). Increased dosage of a *sir-2* gene extends lifespan in *Caenorhabditis elegans*. Nature.

[42] Rogina B., Helfand S.L. (2004). Sir2 mediates longevity in the fly through a pathway related to caloric restriction. Proc. Natl. Acad. Sci. USA.

[43] Howitz K.T., Bitterman K.J., Cohen H.Y., Lamming D.W., Lavu S., Wood J.G., Zipkin R.E., Chung P., Kisielewski A., Zhang L.L. (2003). Small molecule activators of Sirtuins extend *Saccharomycetes cerevisiae* lifespan. Nature.

[44] Bauer J.H., Goupil S., Garber G.B., Helfand S.L. (2004). An accelerated assay for the identification of lifespan-extending interventions in *Drosophila melanogaster*. Proc. Natl. Acad. Sci. USA.

[45] Viswanathan M., Kim S.K., Berdichevsky A., Guarente L. (2005). A role for SIR-2.1 regulation of ER stress response genes in determining *C. elegans* life span. Dev. Cell.

[46] Pearson K.J., Baur J.A., Lewis K.N., Peshkin L., Price N.L., Labinskyy N., Swindell W.R., Kamara D., Minor R.K., Perez E. (2008). Resveratrol delays age-related deterioration and mimics transcriptional aspects of dietary restriction without extending life span. Cell Metab.

[47] Burnett C., Valentini S., Cabreiro F., Goss M., Somogyvári M., Piper M.D., Hoddinott M., Sutphin G.L., Leko V., McElwee J.J. (2011). Absence of effects of Sir2 overexpression on lifespan in *C. elegans* and *Drosophila*. Nature.

[48] Fabrizio P., Gattazzo C., Battistella L., Wei M., Cheng C., McGrew K., Longo V.D. (2005). Sir2 blocks extreme life-span extension. Cell.

[49] Park S.J., Ahmad F., Philp A., Baar K., Williams T., Luo H., Ke H., Rehmann H., Taussig R., Brown A.L. (2012). Resveratrol ameliorates aging-related metabolic phenotypes by inhibiting cAMP phosphodiesterases. Cell.

[50] Gerhart-Hines Z., Dominy J.E., Blättler S.M., Jedrychowski M.P., Banks A.S., Lim J.H., Chim H., Gygi S.P., Puigserver P. (2011). The cAMP/PKA pathway rapidly activates SIRT1 to promote fatty acid oxidation independently of changes in NAD^+^. Mol. Cell.

[51] Kanfi Y., Naiman S., Amir G., Peshti V., Zinman G., Nahum L., Bar-Joseph Z., Cohen H.Y. (2012). The sirtuin SIRT6 regulates lifespan in male mice. Nature.

[52] Tennen R.I., Chua K.F. (2011). Chromatin regulation and genome maintenance by mammalian SIRT6. Trends Biochem. Sci.

[53] Mostoslavsky R., Chua K.F., Lombard D.B., Pang W.W., Fischer M.R., Gellon L., Liu P., Mostoslavsky G., Franco S., Murphy M.M. (2006). Genomic instability and aging-like phenotype in the absence of mammalian SIRT6. Cell.

[54] Guarente L. (2011). Sirtuins, aging, and medicine. N. Engl. J. Med.

[55] Houtkooper R.H., Pirinen E., Auwerx J. (2012). Sirtuins as regulators of metabolism and healthspan. Nat. Rev. Mol. Cell Biol.

[56] Longo V.D., Kennedy B.K. (2006). Sirtuins in aging and age-related disease. Cell.

[57] Baur J.A., Ungvari Z., Minor R.K., Le Couteur D.G., de Cabo R. (2012). Are sirtuins viable targets for improving healthspan and lifespan. Nat. Rev. Drug Disc.

[58] Alcendor R.R., Gao S., Zhai P., Zablocki D., Holle E., Yu X., Tian B., Wagner T., Vatner S.F., Sadoshima J. (2007). Sirt1 regulates aging and resistance to oxidative stress in the heart. Circ. Res.

[59] Brunet A., Sweeney L.B., Sturgill J.F., Chua K.F., Greer P.L., Lin Y., Tran H., Ross S.E., Mostoslavsky R., Cohen H.Y. (2004). Stress-dependent regulation of FOXO transcription factors by the SIRT1 deacetylase. Science.

[60] Van der Horst A., Tertoolen L.G., de Vries-Smits L.M., Frye R.A., Medema R.H., Burgering B.M. (2004). FOXO4 is acetylated upon peroxide stress and deacetylated by the longevity protein hSir2*^SIRT1^*. J. Biol. Chem.

[61] Sengupta A., Molkentin J.D., Paik J.H., DePinho R.A., Yutzey K.E. (2011). FoxO transcription factors promote cardiomyce survival upon induction of oxidative stress. J. Biol. Chem.

[62] Xiong S., Salazar G., Patrushev N., Alexander R.W. (2011). FoxO1 mediates an autofeedback loop regulating SIRT1 expression. J. Biol. Chem.

[63] Yamamoto T., Sadoshima J. (2011). Protection of the heart against ischemia/reperfusion by silent information regulator 1. Trends Cardiovasc. Med.

[64] Yeung F., Hoberg J.E., Ramsey C.S., Keller M.D., Jones D.R., Frye R.A., Mayo M.W. (2004). Modulation of NF-κB-dependent transcription and cell survival by the SIRT1 deacetylase. EMBO J.

[65] Salminen A., Kauppinen A., Suuronen T., Kaarniranta K. (2008). SIRT1 longevity factor suppresses NF-κB-driven immune responses: Regulation of aging via NF-κB acetylation?. BioEssays.

[66] Rajendran R., Garva R., Krstic-Demonacos M., Demonacos C. (2011). Sirtuins: Molecular traffic lights in the crossroad of oxidative stress, chromatin remodeling, and transcription. J. Biomed. Biotechnol.

[67] Salminen A., Hyttinen J.M.T., Kaarniranta K. (2011). AMP-activated protein kinase inhibits NF-κB signaling and inflammation: Impact on healthspan and lifespan. J. Mol. Med.

[68] Sumimoto H. (2008). Structure, regulation and evolution of Nox-family NADPH oxidases that produce reactive oxygen species. FEBS J.

[69] Anrather J., Racchumi G., Iadecola C. (2006). NF-κB regulates phagocytic NADPH oxidase by inducing expression of gp91*^phox^*. J. Biol. Chem.

[70] Manea A., Manea S.A., Gafencu A.V., Raicu M. (2007). Regulation of NADPH oxidase subunit p22(phox) by NF-κB in human aortic smooth muscle cells. Arch. Physiol. Biochem.

[71] Manea A., Tanase L.I., Raicu M., Simionescu M. (2010). Transcriptional regulation of NADPH oxidase isoforms, Nox1 and Nox4, by nuclear factor-κB in human aortic smooth muscle cells. Biochem. Biophys. Res. Commun.

[72] Xie Q., Kashiwabara Y., Nathan C. (1994). Role of transcription factor NF-κB/Rel in induction of nitric oxide synthase. J. Biol. Chem.

[73] Sakitani K., Nishizawa M., Inoue K., Masu Y., Okumura T., Ito S. (1998). Synergistic regulation of inducible nitric oxide synthase gene by CCAAT/enhancer-binding protein β and nuclear factor-κB in hepatocytes. Genes Cells.

[74] Morgan M.J., Liu Z. (2011). Crosstalk of reactive oxygen species and NF-κB signaling. Cell Res.

[75] Kawai Y., Garduno L., Theodore M., Yang J., Arinze I.J. (2011). Acetylation-deacetylation of the transcription factor Nrf2 (nuclear factor erythroid 2-related factor 2) regulates its transcriptional activity and nucleocytoplasmic localization. J. Biol. Chem.

[76] Nguyen T., Sherratt P.J., Pickett C.B. (2003). Regulatory mechanisms controlling gene expression mediated by the antioxidant response element. Annu. Rev. Pharmacol. Toxicol.

[77] Ishii T., Yanagawa T. (2007). Stress-induced peroxiredoxins. Subcell. Biochem.

[78] Tanito M., Agbaga M.P., Anderson R.E. (2007). Upregulation of thioredoxin system via Nrf2-antioxidant responsive element pathway in adaptive-retinal neuroprotection *in vivo* and *in vitro*. Free Radic. Biol. Med.

[79] Zhang D.D., Hannink M. (2003). Distinct cysteine residues in Keap1 are required for Keap1-dependent ubiquitination of Nrf2 and for stabilization of Nrf2 by chemopreventive agents and oxidative stress. Mol. Cell. Biol.

[80] Taguchi K., Fujikawa N., Komatsu M., Ishii T., Unno M., Akaike T., Motohashi H., Yamamoto M. (2012). Keap1 degradation by autophagy for the maintenance of redox homeostasis. Proc. Natl. Acad. Sci. USA.

[81] Komatsu M., Kurokawa H., Waguri S., Taguchi K., Kobayashi A., Ichimura Y., Sou Y.S.., Ueno I., Sakamoto A., Tong K.I. (2010). The selective autophagy substrate p62 activates the stress responsive transcription factor Nrf2 through inactivation of Keap1. Nat. Cell Biol..

[82] Jain A., Lamark T., Sjottem E., Larsen K.B., Awuh J.A., Overvatn A., McMahon M., Hayes J.D., Johansen T. (2010). p62/SQSTM1 is a target gene for transcription factor NRF2 and creates a positive feedback loop by inducing antioxidant response element-driven gene transcription. J. Biol. Chem..

[83] Murphy M.P. (2009). How mitochondria produce reactive oxygen species. Biochem. J.

[84] Fernandez-Marcos P.J., Auwerx J. (2011). Regulation of PGC-1α, a nodal regulator of mitochondrial biogenesis. Am. J. Clin. Nutr.

[85] Scarpulla R.C. (2011). Metabolic control of mitochondrial biogenesis through the PGC-1 family regulatory network. Biochim. Biophys. Acta.

[86] Nemoto S., Fergusson M.M., Finkel T. (2005). SIRT1 functionally interacts with the metabolic regulator and transcriptional coactivator PGC-1α. J. Biol. Chem.

[87] Rodgers J.T., Lerin C., Haas W., Gygi S.P., Spiegelman B.M., Puigserver P. (2005). Nutrient control of glucose homeostasis through a complex of PGC-1α and SIRT1. Nature.

[88] Gurd B.J. (2011). Deacetylation of PGC-1α by SIRT1: Importance for skeletal muscle function and exercise-induced mitochondrial biogenesis. Appl. Physiol. Nutr. Metab.

[89] Philp A., Chen A., Lan D., Meyer G.A., Murphy A.N., Knapp A.E., Olfert I.M., McCurdy C.E., Marcotte G.R., Hogan M.C. (2011). Sirtuin 1 (SIRT1) deacetylase activity is not required for mitochondrial biogenesis or peroxisome proliferator-activated receptor-γ coactivator-1α (PGC-1α) deacetylation following endurance exercise. J. Biol. Chem.

[90] Amat R., Planavila A., Chen S.L., Iglesias R., Giralt M., Villarroya F. (2009). SIRT1 controls the transcription of the peroxisome proliferator-activated receptor-γ co-activator-1α (PGC-1α) gene in skeletal muscle through the PGC-1α autoregulatory loop and interaction with MyoD. J. Biol. Chem.

[91] Brand M.D., Esteves T.C. (2005). Physiological functions of the mitochondrial uncoupling proteins UCP2 and UCP3. Cell Metab.

[92] Mailloux R.J., Harper M.E. (2011). Uncoupling proteins and the control of mitochondrial reactive oxygen species production. Free Radic. Biol. Med.

[93] Andrews Z.B., Horvath T.L. (2009). Uncoupling protein-2 regulates lifespan in mice. Am. J. Physiol. Endocrinol. Metab.

[94] Vidal-Puig A.J., Grujic D., Zhang C.Y., Hagen T., Boss O., Ido Y., Szczepanik A., Wade J., Mootha V., Cortright R. (2000). Energy metabolism in uncoupling protein 3 gene knockout mice. J. Biol. Chem.

[95] Bordone L., Motta M.C., Picard F., Robinson A., Jhala U.S., Apfeld J., McDonagh T., Lemieux M., McBurney M., Szilvasi A. (2006). Sirt1 regulates insulin secretion by repressing UCP2 in pancreatic beta cells. PLoS Biol.

[96] Amat R., Solanes G., Giralt M., Villarroya F. (2007). SIRT1 is involved in glucocorticoids-mediated control of uncoupling protein-3 gene transcription. J. Biol. Chem.

[97] Diano S., Horvath T.L. (2012). Mitochondrial uncoupling protein 2 (UCP2) in glucose and lipid metabolism. Trends Mol. Med.

[98] Kamata H., Honda S., Maeda S., Chang L., Hirata H., Karin M. (2005). Reactive oxygen species promote TNF-α-induced death and sustained JNK activation by inhibiting MAP kinase phosphatases. Cell.

[99] Matsukawa J., Matsuzawa A., Takeda K., Ichijo H. (2004). The ASK1-MAP kinase cascades in mammalian stress response. J. Biochem.

[100] Nasrin N., Kaushik V.K., Fortier E., Wall D., Pearson K.J., de Cabo R., Bordone L. (2009). JNK1 phosphorylates SIRT1 and promotes its enzymatic activity. PLoS One.

[101] Gao Z., Zhang J., Kheterpal I., Kennedy N., Davis R.J., Ye J. (2011). Sirtuin 1 (SIRT1) protein degradation in response to persistent c-Jun *N*-terminal kinase 1 (JNK1) activation contributes to hepatic steatosis in obesity. J. Biol. Chem.

[102] Wang M.C., Bohmann D., Jasper H. (2003). JNK signaling confers tolerance to oxidative stress and extends lifespan in *Drosophila*. Dev Cell.

[103] Biteau B., Karpac J., Hwangbo D., Jasper H. (2011). Regulation of *Drosophila* lifespan by JNK signaling. Exp. Gerontol.

[104] Cardaci S., Filomeni G., Ciriolo M.R. (2012). Redox implications of AMPK-mediated signal transduction beyond energetic clues. J. Cell Sci.

[105] Canto C., Gerhart-Hines Z., Feige J.N., Lagouge M., Noriega L., Molne J.C., Elliott P.J., Puigserver P., Auwerx J. (2009). AMPK regulates energy expenditure by modulating NAD^+^ metabolism and SIRT1 activity. Nature.

[106] Zmijewski J.W., Banerjee S., Bae H., Friggeri A., Lazarowski E.R., Abraham E. (2010). Exposure to hydrogen peroxide induces oxidation and activation of AMP-activated protein kinase. J. Biol. Chem.

[107] Mungai P.T., Waypa G.B., Jairaman A., Prakriya M., Dokic D., Ball M.K., Schumacker P.T. (2011). Hypoxia triggers AMPK activation through reactive oxygen species-mediated activation of calcium release-activated calcium channels. Mol. Cell. Biol.

[108] Emerling B.M., Weinberg F., Snyder C., Burgess Z., Mutlu G.M., Viollet B., Budinger G.R.S., Chandel N.S. (2009). Hypoxic activation of AMPK is dependent on mitochondrial ROS but independent of an increase in AMP/ATP ratio. Free Radic. Biol. Med.

[109] Zhang J., Xie Z., Dong Y., Wang S., Liu C., Zou M.H. (2008). Identification of nitric oxide as an endogenous activator of the AMP-activated protein kinase in vascular endothelial cells. J. Biol. Chem.

[110] Salminen A., Kaarniranta K (2012). AMP-activated protein kinase (AMPK) controls the aging process via an integrated signaling network. Ageing Res. Rev..

[111] Yamakuchi M., Ferlito M., Lowenstein C.J. (2008). miR-34a repression of SIRT1 regulates apoptosis. Proc. Natl. Acad. Sci. USA.

[112] Chen F., Hu S.J. (2012). Effect of microRNAs-34a in cell cycle, differentiation, and apoptosis: A review. J. Biochem. Mol. Toxicol.

[113] Bai X.Y., Ma Y., Ding R., Fu B., Shi S., Chen X.M. (2011). miR-335 and miR-34a promote renal senescence by suppressing mitochondrial antioxidative enzymes. J. Am. Soc. Nephrol.

[114] Li N., Muthusamy S., Liang R., Sarojini H., Wang E. (2011). Increased expression of miR-34a and miR-93 in rat liver during aging, and their impact on the expression of Mgst1 and Sirt1. Mech. Ageing Dev.

[115] Ito T., Yagi S., Yamakuchi M. (2010). MicroRNA-34a regulation of endothelial senescence. Biochem. Biophys. Res. Commun.

[116] Liu B., Chen Y., St. Clair D.K. (2008). ROS and p53: A versatile partnership. Free Radic. Biol. Med.

[117] Ladelfa M.F., Toledo M.F., Laiseca J.E., Monte M. (2011). Interaction of p53 with tumor suppressive and oncogenic signaling pathways to control cellular reactive oxygen species production. Antioxid. Redox Signal.

[118] Guo Z., Kozlov S., Lavin M.F., Person M.D., Paull T.T. (2010). ATM activation by oxidative stress. Science.

[119] Maillet A., Pervaiz S. (2012). Redox regulation of p53, redox effectors regulated by p53: A subtle balance. Antioxid. Redox Signal.

[120] Furukawa A., Tada-Oikawa S., Kawanishi S., Oikawa S. (2007). H_2_O_2_ accelerates cellular senescence by accumulation of acetylated p53 via decrease in the function of SIRT1 by NAD^+^ depletion. Cell. Physiol. Biochem.

[121] Caito S., Rajendrasozhan S., Cook S., Chung S., Yao H., Friedman A.E., Brookes P.S., Rahman I. (2010). SIRT1 is a redox-sensitive deacetylase that is post-translationally modified by oxidants and carbonyl stress. FASEB J.

[122] Cai W., Ramdas M., Zhu L., Chen X., Striker G.E., Vlassara H. (2012). Oral advanced glycation endproducts (AGEs) promote insulin resistance and diabetes by depleting the antioxidant defenses AGE receptor-1 and sirtuin 1. Proc. Natl. Acad. Sci. USA.

[123] Rajendrasozhan S., Yang S.R., Kinnula V.L., Rahman I. (2008). SIRT1, an antiinflammatory and antiaging protein, is decreased in lungs of patients with chronic obstructive pulmonary disease. Am. J. Respir. Crit. Care Med.

[124] Zee R.S., Yoo C.B., Pimentel D.R., Perlman D.H., Burgoyne J.R., Hou X., McComb M.E., Costello C.E., Cohen R.A., Bachschmid M.M. (2010). Redox regulation of Sirtuin-1 by *S*-glutathiolation. Antioxid. Redox Signal.

[125] Kornberg M.D., Sen N., Hara M.R., Juluri K.R., Nguyen J.V., Snowman A.M., Law L., Hester L.D., Snyder S.H. (2010). GAPDH mediates nitrosylation of nuclear proteins. Nat. Cell Biol.

[126] Tong C., Morrison A., Mattison S., Qian S., Bryniarski M., Rankin B., Wang J., Thomas D.P., Li J (2013). Impaired SIRT1 nucleocytoplasmic shuttling in the senescent heart during ischemic stress. FASEB J..

[127] Yang Y., Fu W., Chen J., Olashaw N., Zhang X., Nicosia S.V., Bhalla K., Bai W. (2007). SIRT1 sumoylation regulates its deacetylase activity and cellular response to genotoxic stress. Nat. Cell Biol.

[128] Cuervo A.M. (2008). Autophagy and aging: Keeping that old broom working. Trends Genet.

[129] De Magalhaes J.P., Curado J., Church G.M. (2009). Meta-analysis of age-related gene expression profiles identifies common signatures of aging. Bioinformatics.

[130] Salminen A., Kaarniranta K. (2009). NF-κB signaling in the aging process. J. Clin. Immunol.

[131] Cannizzo E.S., Clement C.C., Sahu R., Follo C., Santambrogio L. (2011). Oxidative stress, inflammaging and immunosenescence. J. Proteomics.

[132] Mizushima N., Klionsky D.J. (2007). Protein turnover via autophagy: Implications for metabolism. Annu. Rev. Nutr.

[133] Mizushima N., Komatsu M. (2011). Autophagy: Renovation of cells and tissues. Cell.

[134] Lee I.H., Cao L., Mostoslavsky R., Lombard D.B., Liu J., Bruns N.E., Tsokos M., Alt F.W., Finkel T. (2008). A role for the NAD-dependent deacetylase Sirt1 in the regulation of autophagy. Proc. Natl. Acad. Sci. USA.

[135] Wang P., Guan Y.F., Du H., Zhai Q.W., Su D.F., Miao C.Y. (2012). Induction of autophagy contributes to the neuroprotection of nicotinamide phosphoribosyltransferase in cerebral ischemia. Autophagy.

[136] Hariharan N., Maejima Y., Nakae J., Paik J., DePinho R.A., Sadoshima J. (2010). Deacetylation of FoxO by Sirt1 plays an essential role in mediating starvation-induced autophagy in cardiac myocytes. Circ. Res.

[137] Hyttinen J.M.T., Niittykoski M., Salminen A., Kaarniranta K. (2013). Maturation of autophagosomes and endosomes: A key role for Rab7. Biochim. Biophys. Acta.

[138] Mammucari C., Milan G., Romanello V., Masiero E., Rudolf R., Del Piccolo P., Burden S.J., Di Lisi R., Sandri C., Zhao J. (2007). FoxO3 controls autophagy in skeletal muscle *in vivo*. Cell Metab.

[139] Nadtochiy S.M., Yao H., McBurney M.W., Gu W., Guarente L., Rahman I., Brookes P.S. (2011). SIRT1-mediated acute cardioprotection. Am. J. Physiol. Heart Circ. Physiol.

[140] Jeong J.K., Moon M.H., Lee Y.J., Seol J.W., Park S.Y. (2013). Autophagy induced by the class III histone deacetylase Sirt1 prevents prion peptide neurotoxicity. Neurobiol. Aging.

[141] Gurd B.J., Yoshida Y., Lally J., Holloway G.P., Bonen A. (2009). The deacetylase enzyme SIRT1 is not associated with oxidative capacity in rat heart and skeletal muscle and its overexpression reduces mitochondrial biogenesis. J. Physiol.

[142] Kawashima T., Inuzuka Y., Okuda J., Kato T., Niizuma S., Tamaki Y., Iwanaga Y., Kawamoto A., Narazaki M., Matsuda T. (2011). Constitutive SIRT1 overexpression impairs mitochondria and reduces cardiac function in mice. J. Mol. Cell. Cardiol.

[143] Huang J., Lam G.Y., Brumell J.H. (2011). Autophagy signaling through reactive oxygen species. Antioxid. Redox Signal.

[144] Scherz-Shouval R., Elazar Z. (2011). Regulation of autophagy by ROS: Physiology and pathology. Trends Biochem. Sci.

[145] Lee J., Giordano S., Zhang J. (2012). Autophagy, mitochondria and oxidative stress: Cross-talk and redox signalling. Biochem. J.

[146] Chen Y., Azad M.B., Gibson S.B. (2009). Superoxide is the major reactive oxygen species regulating autophagy. Cell Death Differ.

[147] Scherz-Shouval R., Shvets E., Fass E., Shorer H., Gil L., Elazar Z. (2007). Reactive oxygen species are essential for autophagy and specifically regulate the activity of Atg4. EMBO J.

[148] Wei Y., Pattingre S., Sinha S., Bassik M., Levine B. (2008). JNK1-mediated phosphorylation of Bcl-2 regulates starvation-induced autophagy. Mol. Cell.

[149] Park K.J., Lee S.H., Lee C.H., Jang J.Y., Chung J., Kwon M.H., Kim Y.S. (2009). Upregulation of Beclin-1 expression and phosphorylation of Bcl-2 and p53 are involved in the JNK-mediated autophagic cell death. Biochem. Biophys. Res. Commun..

[150] Zhang X.Y., Wu X.Q., Deng R., Sun T., Feng G.K., Zhu X.F. (2013). Upregulation of Sestrin 2 expression via JNK pathway activation contributes to autophagy induction in cancer cells. Cell Signal.

[151] Xu P., Das M., Reilly J., Davis R.J. (2011). JNK regulates FoxO-dependent autophagy in neurons. Genes Dev.

[152] Sarkar S., Korolchuk V.I., Renna M., Imarisio S., Fleming A., Williams A., Garcia-Arencibia M., Rose C., Luo S., Underwood B.R. (2011). Complex inhibitory effects of nitric oxide on autophagy. Mol. Cell.

[153] Alexander A., Cai S.L., Kim J., Nanez A., Sahin M., MacLean K.H., Inoki K., Guan K.L., Shen J., Person M.D. (2010). ATM signals to TSC2 in the cytoplasm to regulate mTORC1 in response to ROS. Proc. Natl. Acad. Sci. USA.

[154] Li L., Chen Y., Gibson S.B. (2013). Starvation-induced autophagy is regulated by mitochondrial reactive oxygen species leading to AMPK activation. Cell Signal.

[155] McClung J.M., Judge A.R., Powers S.K., Yan Z. (2010). p38 MAPK links oxidative stress to autophagy-related gene expression in cachectic muscle wasting. Am. J. Physiol. Cell. Physiol.

[156] Luo Y., Zou P., Zou J., Wang J., Zhou D., Liu L. (2011). Autophagy regulates ROS-induced cellular senescence via p21 in a p38 MAPKα dependent manner. Exp. Gerontol.

[157] Lee J.W., Park S., Takahashi Y., Wang H.G. (2010). The association of AMPK with ULK1 regulates autophagy. PLoS One.

[158] Sanchez A.M., Csibi A., Raibon A., Cornille K., Gay S., Bernardi H., Candau R. (2012). AMPK promotes skeletal muscle autophagy through activation of forkhead FoxO3a and interaction with Ulk1. J. Cell. Biochem.

[159] Bensimon A., Aebersold R., Shiloh Y. (2011). Beyond ATM: The protein kinase landscape of the DNA damage response. FEBS Lett.

[160] Wang Y., Nartiss Y., Steipe B., McQuibban G.A., Kim P.K. (2012). ROS-induced mitochondrial depolarization initiates PARK2/PARKIN-dependent mitochondrial degradation by autophagy. Autophagy.

[161] Vallabhapurapu S., Karin M. (2009). Regulation and function of NF-κB transcription factors in the immune system. Annu. Rev. Immunol.

[162] Karin M., Lin A. (2002). NF-κB at the crossroads of life and death. Nat. Immunol.

[163] Perkins N.D. (2007). Integrating cell-signalling pathways with NF-κB and IKK function. Nat. Rev. Mol. Cell Biol.

[164] Johnson R.F., Perkins N.D. (2012). Nuclear factor-κB, p53, and mitochondria: Regulation of cellular metabolism and the Warburg effect. Trends Biochem. Sci.

[165] Helenius M., Hänninen M., Lehtinen S.K., Salminen A. (1996). Changes associated with aging and replicative senescence in the regulation of transcription factor nuclear factor-κB. Biochem. J.

[166] Helenius M., Kyrylenko S., Vehviläinen P., Salminen A. (2001). Characterization of aging-associated up-regulation of constitutive nuclear factor-κB binding activity. Antioxid. Redox Signal.

[167] Adler A.S., Sinha S., Kawahara T.L., Zhang J.Y., Segal E., Chang H.Y. (2007). Motif module map reveals enforcement of aging by continual NF-κB activity. Genes Dev.

[168] Csiszar A., Wang M., Lakatta E.G., Ungvari Z. (2008). Inflammation and endothelial dysfunction during aging: Role of NF-κB. J. Appl. Physiol.

[169] Cribbs D.H., Berchtold N.C., Perreau V., Coleman P.D., Rogers J., Tenner A.J., Cotman C.W. (2012). Extensive innate immune gene activation accompanies brain aging, increasing vulnerability to cognitive decline and neurodegeneration: A microarray study. J. Neuroinflamm.

[170] Lin L., Hron J.D., Peng S.L. (2004). Regulation of NF-κB, Th activation, and autoinflammation by the forkhead transcription factor Foxo3a. Immunity.

[171] Gillum M.P., Kotas M.E., Erion D.M., Kursawe R., Chatterjee P., Nead K.T., Muise E.S., Hsiao J.J., Frederick D.W., Yonemitsu S. (2011). SirT1 regulates adipose tissue inflammation. Diabetes.

[172] Liu T.F., Yoza B.K., Gazzar M.E., Vachharajani V.T., McCall C.E. (2011). NAD^+^-dependent SIRT1 deacetylase participates in epigenetic reprogramming during endotoxin tolerance. J. Biol. Chem.

[173] Peters T., Weiss J.M., Sindrilaru A., Wang H., Oreshkova T., Wlaschek M., Maity P., Reimann J., Scharffetter-Kochanek K. (2009). Reactive oxygen intermediate-induced pathomechanisms contribute to immunosenescence, chronic inflammation and autoimmunity. Mech. Ageing Dev.

[174] Otani H. (2011). Oxidative stress as pathogenesis of cardiovascular risk associated with metabolic syndrome. Antioxid. Redox Signal.

[175] Gloire G., Legrand-Poels S., Piette J. (2006). NF-κB activation by reactive oxygen species: Fifteen years later. Biochem. Pharmacol.

[176] Zhou R., Tardivel A., Thorens B., Choi I., Tschopp J. (2010). Thioredoxin-interacting protein links oxidative stress to inflammasome activation. Nat. Immunol.

[177] Rahman I., Biswas S.K., Kirkham P.A. (2006). Regulation of inflammation and redox signalling by dietary polyphenols. Biochem. Pharmacol.

[178] Salminen A., Lehtonen M., Suuronen T., Kaarniranta K., Huuskonen J. (2008). Terpenoids: Natural inhibitors of NF-κB signaling with anti-inflammatory and anticancer potential. Cell. Mol. Life Sci.

[179] Salminen A., Hyttinen J.M., Kauppinen A., Kaarniranta K. (2012). Context-dependent regulation of autophagy by IKK-NF-κB signaling: Impact on the aging process. Int. J. Cell Biol.

[180] Kenyon C. (2011). The first long-lived mutants: Discovery of the insulin/IGF-1 pathway for ageing. Phil. Trans. R. Soc. B.

[181] Braeckman B.P., Vanfleteren J.R. (2007). Genetic control of longevity in *C. elegans*. Exp. Gerontol.

[182] Bartke A (2011). Pleiotropic effects of growth hormone signaling in aging. Trends Endocrinol. Metab.

[183] Brown-Borg H.M. (2009). Hormonal control of aging in rodents: The somatotropic axis. Mol. Cell. Endocrinol.

[184] Bartke A., Brown-Borg H. (2004). Life extension in the dwarf mouse. Curr. Top. Dev. Biol.

[185] Lithgow G.J., Walker G.A. (2002). Stress resistance as a determinate of *C. elegans* lifespan. Mech. Ageing Dev.

[186] Salminen A., Kaarniranta K. (2010). Insulin/IGF-1 paradox of aging: Regulation via AKT/IKK/NF-κB signaling. Cell. Signal.

[187] Iwasaki Y., Nishiyama M., Taguchi T., Asai M., Yoshida M., Kambayashi M., Terada Y., Hashimoto K. (2009). Insulin exhibits short-term anti-inflammatory but long-term proinflammatory effects *in vitro*. Mol. Cell. Endocrinol.

[188] Arkan M.C., Hevener A.L., Greten F.R., Maeda S., Li Z.W., Long J.M., Wynshaw-Boris A., Poli G., Olefsky J., Karin M. (2005). IKK-β links inflammation to obesity-induced insulin resistance. Nat. Med.

[189] Musarò A., Dobrowolny G., Rosenthal N. (2007). The neuroprotective effects of a locally acting IGF-1 isoform. Exp. Gerontol.

[190] Pelosi L., Giacinti C., Nardis C., Borsellino G., Rizzuto E., Nicoletti C., Wannenes F., Battistini L., Rosenthal N., Molinaro M. (2007). Local expression of IGF-1 accelerates muscle regeneration by rapidly modulating inflammatory cytokines and chemokines. FASEB J.

[191] Vinciguerra M., Santini M.P., Claycomb W.C., Ladurner A.G., Rosenthal N. (2010). LocalIGF-1isoformprotects cardiomyocytes from hypertrophic and oxidative stresses via SirT1 activity. Aging (Albany NY).

[192] Bolasco G., Calogero R., Carrara M., Banchaabouchi M.A., Bilbao D., Mazzoccoli G., Vinciguerra M. (2012). Cardioprotective mIGF-1/SIRT1 signaling induces hypertension, leukocytosis and fear response in mice. Aging (Albany NY).

[193] Vinciguerra M., Santini M.P., Martinez C., Pazienza V., Claycomb W.C., Giuliani A., Rosenthal N. (2012). mIGF-1/JNK1/SirT1 signaling confers protection against oxidative stress in the heart. Aging Cell.

[194] Tang B.L. (2006). SIRT1, neuronal cell survival and the insulin/IGF-1 aging paradox. Neurobiol. Aging.

[195] Li Y., Xu W., McBurney M.W., Longo V.D. (2008). SirT1 inhibition reduces IGF-I/IRS-2/Ras/ERK1/2 signaling and protects neurons. Cell Metab.

[196] Wu D., Qiu Y., Gao X., Yuan X.B., Zhai Q. (2011). Overexpression of SIRT1 in mouse forebrain impairs lipid/glucose metabolism and motor function. PLoS One.

[197] Zhang J (2007). The direct involvement of SirT1 in insulin-induced insulin receptor substrate-2 tyrosine phosphorylation. J. Biol. Chem.

